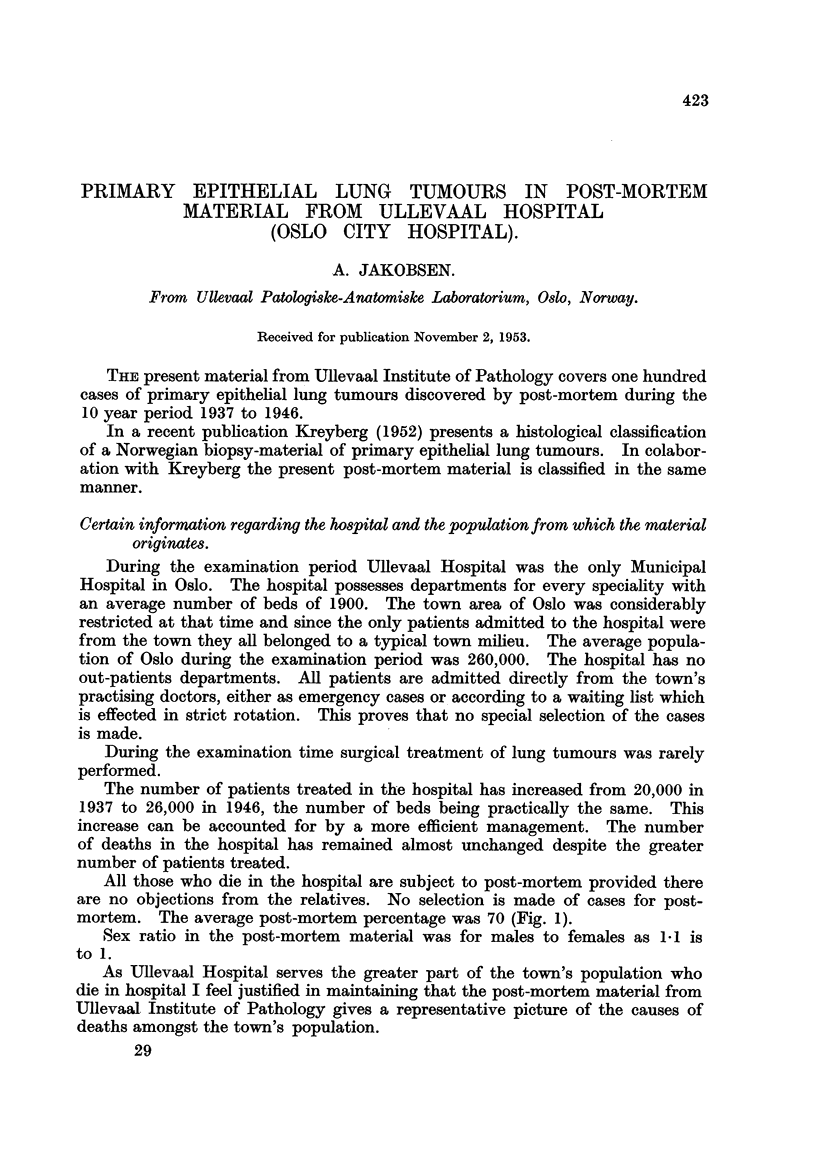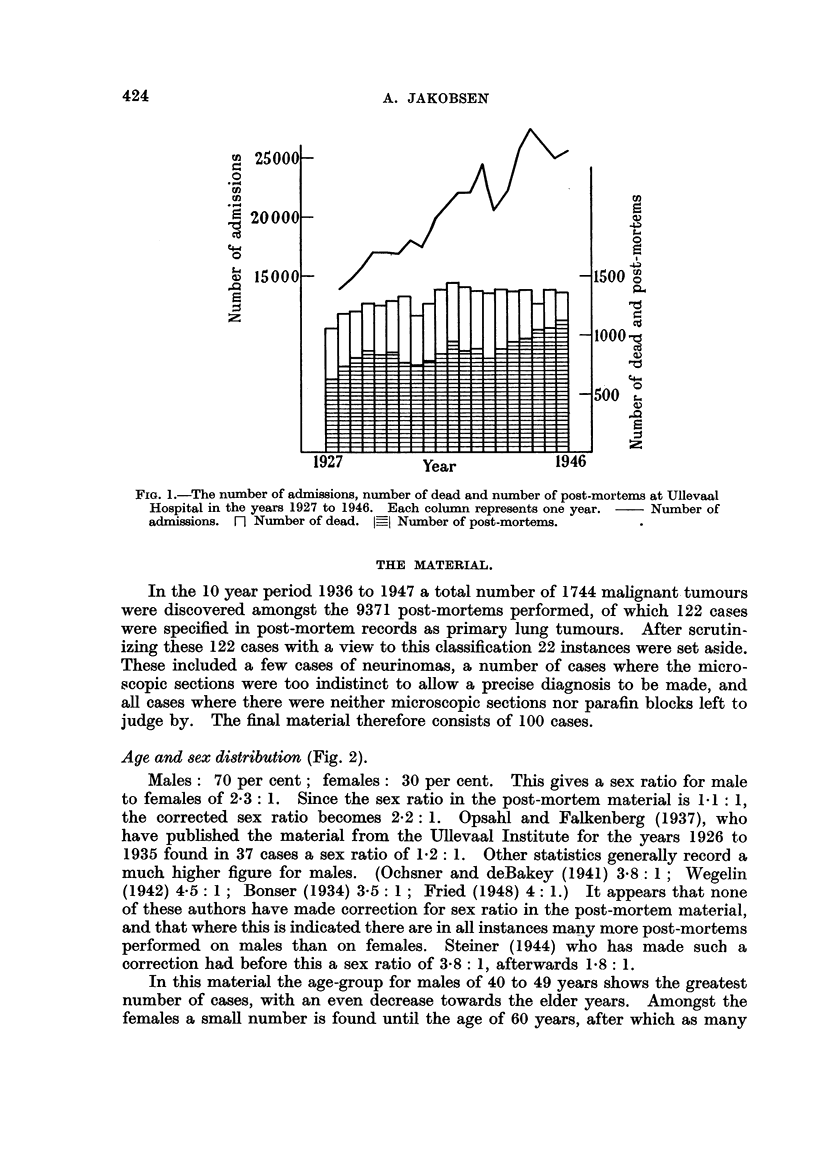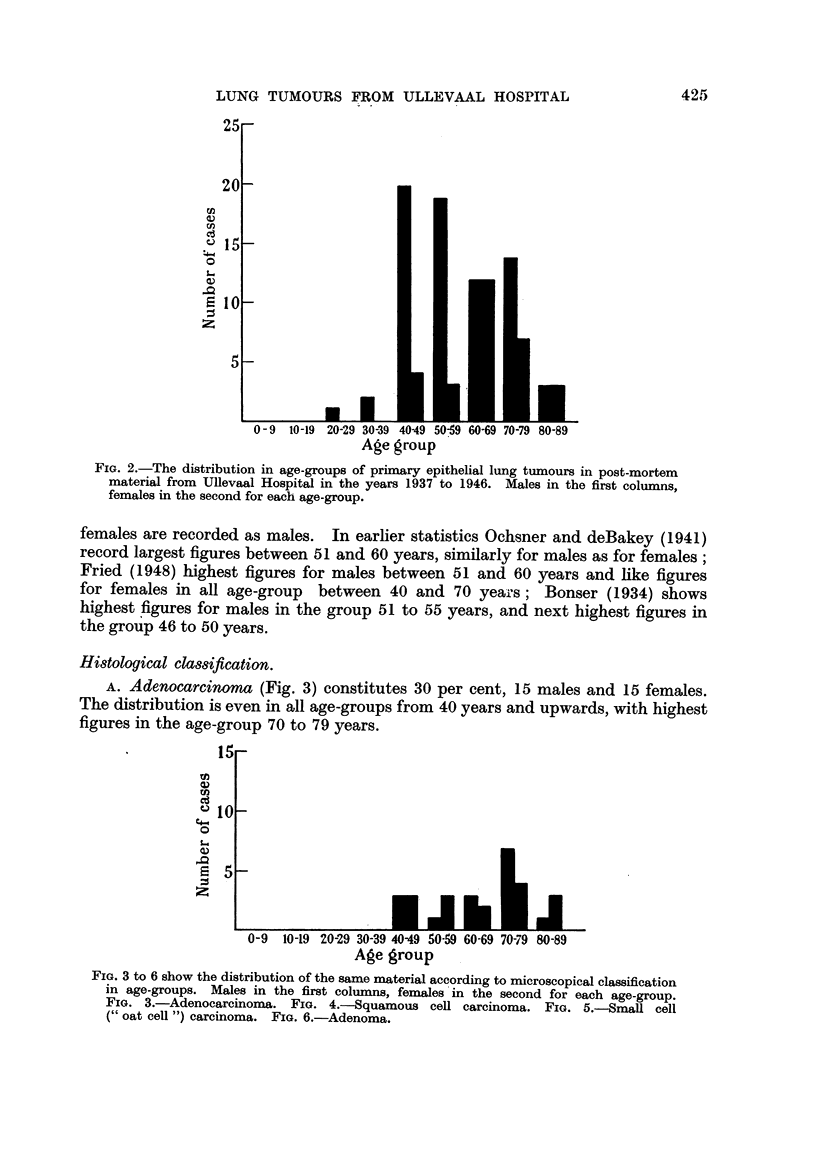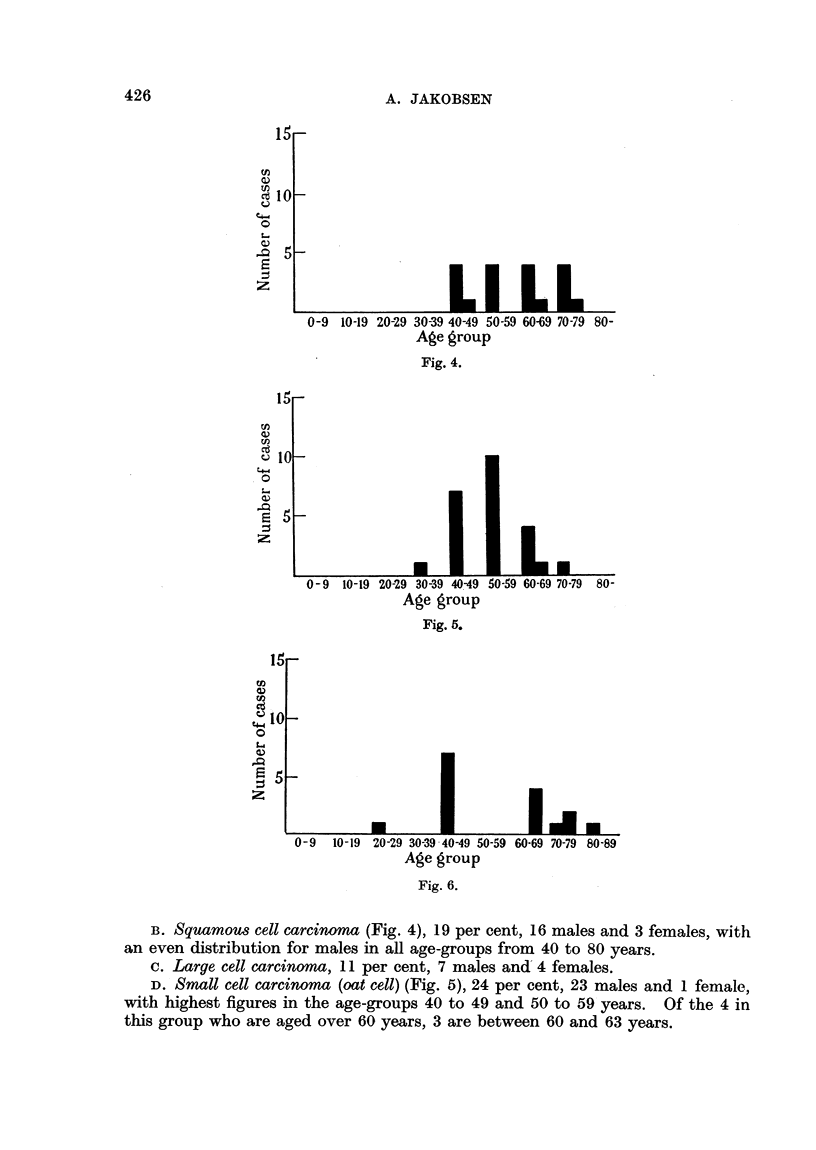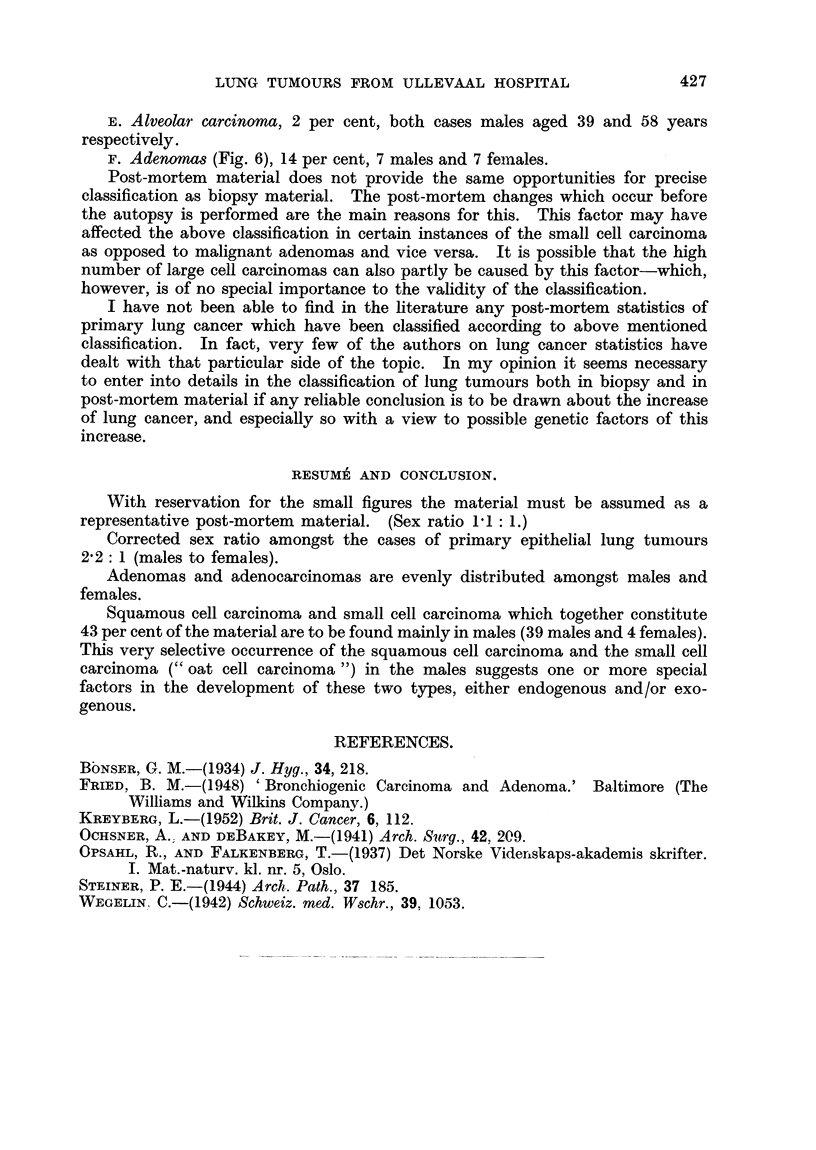# Primary Epithelial Lung Tumours in Post-Mortem Material from Ullevaal Hospital (Oslo City Hospital)

**DOI:** 10.1038/bjc.1953.41

**Published:** 1953-12

**Authors:** A. Jakobsen


### PRIMARY EPITHELIAL LUNG TUMOURS IN POST-MORTEM MATERIAL FROM ULLEVAAL HOSPITAL (OSLO CITY HOSPITAL).

A. JAKOBSEN.

*From Ullevaal Patologiske-Anatomiske Laboratorium, Oslo, Norway.*

Received for publication November 2, 1953.

THE present material from Ullevaal Institute of Pathology covers one hundred cases of primary epithelial lung tumours discovered by post-mortem during the 10 year period 1937 to 1946.

In a recent publication Kreyberg (1952) presents a histological classification of a Norwegian biopsy-material of primary epithelial lung tumours. In colaboration with Kreyberg the present post-mortem material is classified in the same manner.

*Certain information regarding the hospital and the population from which the material originates.*

During the examination period Ullevaal Hospital was the only Municipal Hospital in Oslo. The hospital possesses departments for every speciality with an average number of beds of 1900. The town area of Oslo was considerably restricted at that time and since the only patients admitted to the hospital were from the town they all belonged to a typical town milieu. The average population of Oslo during the examination period was 260,000. The hospital has no out-patients departments. All patients are admitted directly from the town's practising doctors, either as emergency cases or according to a waiting list which is effected in strict rotation. This proves that no special selection of the cases is made.

During the examination time surgical treatment of lung tumours was rarely performed.

The number of patients treated in the hospital has increased from 20,000 in 1937 to 26,000 in 1946, the number of beds being practically the same. This increase can be accounted for by a more efficient management. The number of deaths in the hospital has remained almost unchanged despite the greater number of patients treated.

All those who die in the hospital are subject to post-mortem provided there are no objections from the relatives. No selection is made of cases for post-mortem. The average post-mortem percentage was 70 (Fig. 1).

Sex ratio in the post-mortem material was for males to females as 1·1 is to 1.

As Ullevaal Hospital serves the greater part of the town's population who die in hospital I feel justified in maintaining that the post-mortem material from Ullevaal Institute of Pathology gives a representative picture of the causes of deaths amongst the town's population.

Fig. 1.—The number of admissions, number of dead and number of post-mortems at Ullevaal Hospital in the years 1927 to 1946. Each column represents one year. —— Number of admissions. ⊓ Number of dead. |≡| Number of post-mortems.

THE MATERIAL.

In the 10 year period 1936 to 1947 a total number of 1744 malignant tumours were discovered amongst the 9371 post-mortems performed, of which 122 cases were specified in post-mortem records as primary lung tumours. After scrutinizing these 122 cases with a view to this classification 22 instances were set aside. These included a few cases of neurinomas, a number of cases where the microscopic sections were too indistinct to allow a precise diagnosis to be made, and all cases where there were neither microscopic sections nor parafin blocks left to judge by. The final material therefore consists of 100 cases.

*Age and sex distribution* (Fig. 2).

Males : 70 per cent ; females : 30 per cent. This gives a sex ratio for male to females of 2·3 : 1. Since the sex ratio in the post-mortem material is 1·1 : 1, the corrected sex ratio becomes 2·2 : 1. Opsahl and Falkenberg (1937), who have published the material from the Ullevaal Institute for the years 1926 to 1935 found in 37 cases a sex ratio of 1·2 : 1. Other statistics generally record a much higher figure for males. (Ochsner and deBakey (1941) 3·8 : 1 ; Wegelin (1942) 4·5 : 1 ; Bonser (1934) 3·5 : 1 ; Fried (1948) 4 : 1.) It appears that none of these authors have made correction for sex ratio in the post-mortem material, and that where this is indicated there are in all instances many more post-mortems performed on males than on females. Steiner (1944) who has made such a correction had before this a sex ratio of 3·8 : 1, afterwards 1·8 : 1.

In this material the age-group for males of 40 to 49 years shows the greatest number of cases, with an even decrease towards the elder years. Amongst the females a small number is found until the age of 60 years, after which as many

FIG. 2.—The distribution in age-groups of primary epithelial lung tumours in post-mortem material from Ullevaal Hospital in the years 1937 to 1946. Males in the first columns, females in the second for each age-group.

females are recorded as males. In earlier statistics Ochsner and deBakey (1941) record largest figures between 51 and 60 years, similarly for males as for females ; Fried (1948) highest figures for males between 51 and 60 years and like figures for females in all age-group between 40 and 70 years ; Bonser (1934) shows highest figures for males in the group 51 to 55 years, and next highest figures in the group 46 to 50 years.

*Histological classification.*

A. *Adenocarcinoma* (Fig. 3) constitutes 30 per cent, 15 males and 15 females. The distribution is even in all age-groups from 40 years and upwards, with highest figures in the age-group 70 to 79 years.

FIG. 3 to 6 show the distribution of the same material according to microscopical classification in age-groups. Males in the first columns, females in the second for each age-group. FIG. 3.—Adenocarcinoma. FIG. 4.—Squamous cell carcinoma. FIG. 5.—Small cell (" oat cell ") carcinoma. FIG. 6.—Adenoma.

Fig. 4.

Fig. 5.

Fig. 6.

B. *Squamous cell carcinoma* (Fig. 4), 19 per cent, 16 males and 3 females, with an even distribution for males in all age-groups from 40 to 80 years.

C. *Large cell carcinoma,* 11 per cent, 7 males and 4 females.

D. *Small cell carcinoma* (*oat cell*) (Fig. 5), 24 per cent, 23 males and 1 female, with highest figures in the age-groups 40 to 49 and 50 to 59 years. Of the 4 in this group who are aged over 60 years, 3 are between 60 and 63 years.

E. *Alveolar carcinoma*, 2 per cent, both cases males aged 39 and 58 years respectively.

F. *Adenomas* (Fig. 6), 14 per cent, 7 males and 7 females.

Post-mortem material does not provide the same opportunities for precise classification as biopsy material. The post-mortem changes which occur before the autopsy is performed are the main reasons for this. This factor may have affected the above classification in certain instances of the small cell carcinoma as opposed to malignant adenomas and vice versa. It is possible that the high number of large cell carcinomas can also partly be caused by this factor—which, however, is of no special importance to the validity of the classification.

I have not been able to find in the literature any post-mortem statistics of primary lung cancer which have been classified according to above mentioned classification. In fact, very few of the authors on lung cancer statistics have dealt with that particular side of the topic. In my opinion it seems necessary to enter into details in the classification of lung tumours both in biopsy and in post-mortem material if any reliable conclusion is to be drawn about the increase of lung cancer, and especially so with a view to possible genetic factors of this increase.

### RESUMÉ AND CONCLUSION.

With reservation for the small figures the material must be assumed as a representative post-mortem material. (Sex ratio 1·1 : 1.)

Corrected sex ratio amongst the cases of primary epithelial lung tumours 2·2 : 1 (males to females).

Adenomas and adenocarcinomas are evenly distributed amongst males and females.

Squamous cell carcinoma and small cell carcinoma which together constitute 43 per cent of the material are to be found mainly in males (39 males and 4 females). This very selective occurrence of the squamous cell carcinoma and the small cell carcinoma (" oat cell carcinoma ") in the males suggests one or more special factors in the development of these two types, either endogenous and/or exogenous.

### REFERENCES.

BONSER, G. M.—(1934) *J. Hyg.*, **34**, 218.

FRIED, B. M.—(1948) 'Bronchiogenic Carcinoma and Adenoma.' Baltimore (The Williams and Wilkins Company.)

KREYBERG, L.—(1952) *Brit. J. Cancer*, **6**, 112.

OCHSNER, A., AND DEBAKEY, M.—(1941) *Arch. Surg.*, **42**, 209.

OPSAHL, R., AND FALKENBERG, T.—(1937) Det Norske Videnskaps-akademis skrifter. I. Mat.-naturv. kl. nr. 5, Oslo.

STEINER, P. E.—(1944) *Arch. Path.*, **37** 185.

WEGELIN, C.—(1942) *Schweiz. med. Wschr.*, **39**, 1053.